# Medicare Spending on Drugs With Accelerated Approval, 2015-2019

**DOI:** 10.1001/jamahealthforum.2021.3937

**Published:** 2021-12-03

**Authors:** Benjamin N. Rome, William B. Feldman, Aaron S. Kesselheim

**Affiliations:** 1 Program On Regulation, Therapeutics, And Law (PORTAL), Division of Pharmacoepidemiology and Pharmacoeconomics, Department of Medicine, Brigham and Women’s Hospital, Boston, Massachusetts; 2 Harvard Medical School, Boston, Massachusetts; 3 Division of Pulmonary and Critical Care Medicine, Department of Medicine, Brigham and Women’s Hospital, Boston, Massachusetts

## Abstract

This article examines the budgetary implications of high-priced accelerated approval drugs by estimating Medicare spending on these drugs from 2015 through 2019.

## Introduction

Under the accelerated approval program, the US Food and Drug Administration (FDA) approves drugs that treat serious or life-threatening conditions if their pivotal trials show changes to surrogate measures that are reasonably likely to predict actual clinical outcomes. Drugs granted accelerated approval must undergo confirmatory studies, but in some cases these studies have not been completed in a timely fashion,^1^ and the FDA has had difficulty withdrawing drugs or removing indications even when confirmatory studies fail to demonstrate clinical benefit.^2^ Although drugs made available via accelerated approval have no proven clinical benefit, manufacturers still charge high prices.^3^ To understand the budgetary implications of high-priced accelerated approval drugs, we estimated Medicare spending on these drugs from 2015 through 2019.

## Methods

We identified drugs that were granted accelerated approval for 1 or more indications through December 2019.^4^ We obtained annual Medicare Part B and D spending between 2015 and 2019 from publicly available Drug Spending Dashboards. For Medicare Part D, we estimated net spending by subtracting estimated average annual rebates and other discounts for specific drug classes based on data from a recent federal report.^5^ In a sensitivity analysis, we alternatively used estimated drug-specific rebates from SSR Health. Part B spending data already reflect discounts, but we adjusted spending to account for missing data for 7 newly marketed drugs (see eMethods, eTable 1, and eTable 2 in Supplement).

We stratified spending based on periods when drugs were exclusively approved via the accelerated approval pathway vs periods when drugs were approved for multiple indications via both the accelerated and traditional approval pathways, prorating annual spending estimates as necessary. We compared spending on accelerated approval drugs with total estimated net Medicare Part B and D spending.^5,6^ This study follows Consolidated Health Economic Evaluation Reporting Standards (CHEERS) reporting guidelines for economic analyses and was not submitted for institutional review board approval because it used public, nonidentifiable data (45 CFR 46.102).

## Results

**Figure 1.**
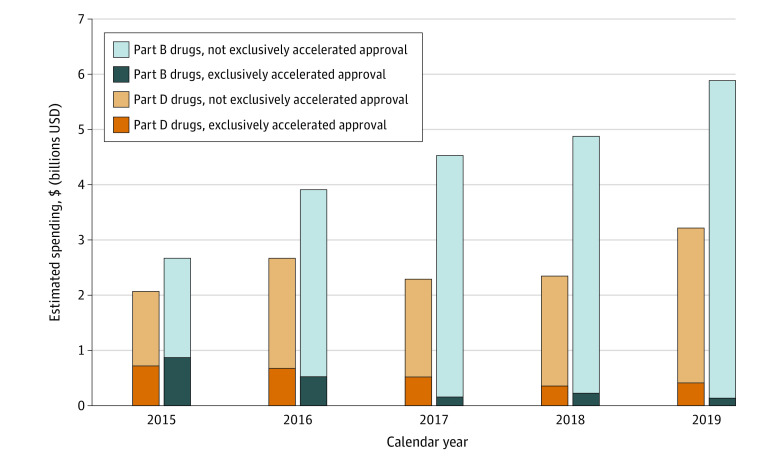
Estimated Medicare Spending on Accelerated Approval Drugs, 2015-2019 Estimated annual Medicare spending on drugs with an accelerated approval indication reimbursed under Part B (blue) and Part D (orange) was stratified based on time when a drug was exclusively marketed for an accelerated approval indication vs time when a drug’s labeling additionally included one or more indications via the traditional approval pathway. All spending data were converted to 2020 US dollars using the Consumer Price Index for All Urban Consumers, and mid-year changes in accelerated approval status were prorated. Part D drug spending estimates are net of estimated class-specific rebates and discounts and include traditional Medicare and Medicare Advantage beneficiaries; Part B drug spending excludes Medicare Advantage beneficiaries and estimates were adjusted to account for missing data in the first years a drug is marketed.

A total of 66 drugs with Medicare spending from 2015 through 2019 had at least 1 accelerated approval indication. Among these, 49 (74%) were oncologic drugs. Annual spending on all drugs with an accelerated approval indication increased from 2015 to 2019 (Part D: $2.1 to $3.2 billion; Part B: $2.7 to $5.9 billion), but spending on drugs with only accelerated approval indications decreased (Part D: $0.7 to $0.4 billion; Part B: $0.9 to $0.1 billion; Figure 1). In a sensitivity analysis using SSR Health rebates, estimated Part D spending was similar ($3.6 billion for all accelerated approval drugs and $0.5 billion for exclusively accelerated approval drugs in 2019).

**Figure 2.**
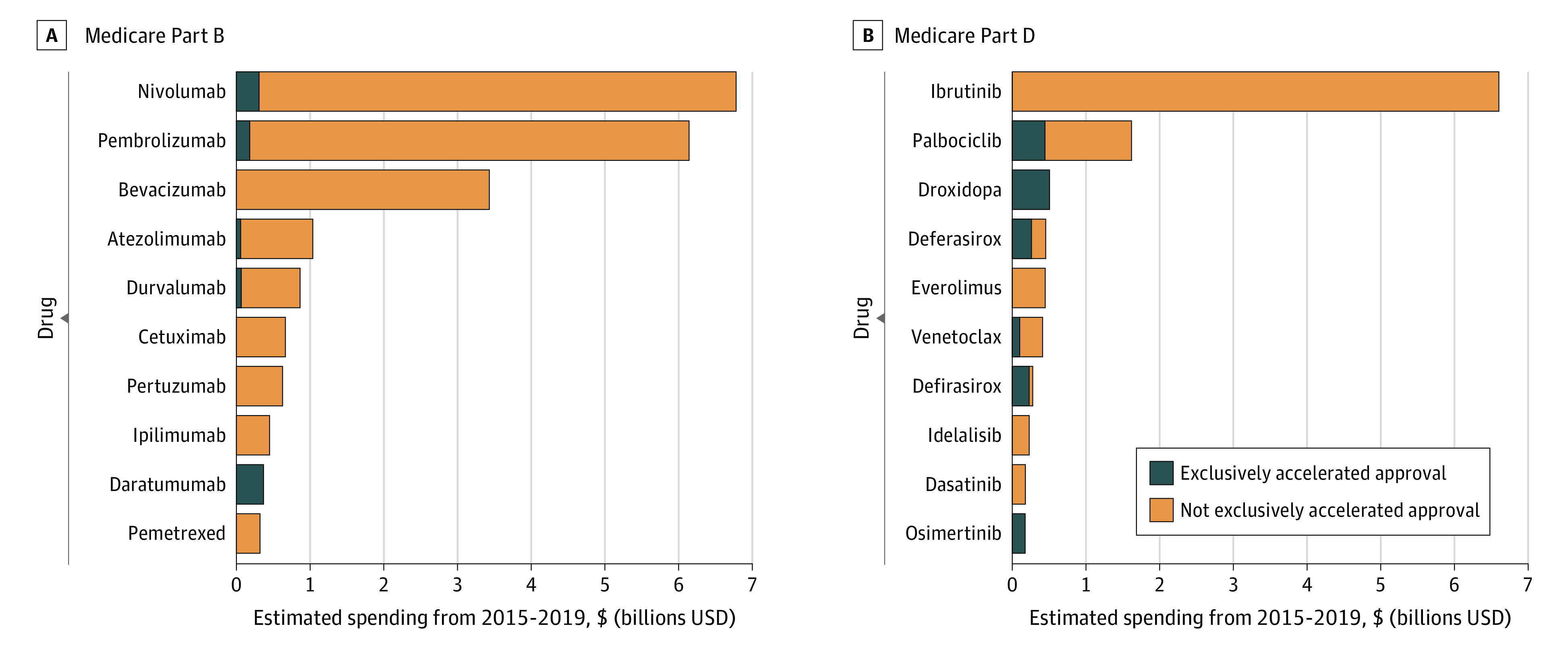
Accelerated Approval Drugs With the Highest Medicare Spending, 2015-2019 Estimated spending for the top 10 Medicare Part B drugs (A) and Part D drugs (B) during the time from 2015 through 2019 that a drug had an accelerated approval indication on the label, including time when the drug was marketed exclusively via accelerated approval (blue) or time when the drug had other labeled indications via traditional US Food and Drug Administration approval pathways (orange).

The Part B accelerated approval drugs with the highest spending during the 5-year study period were nivolumab ($6.8 billion), pembrolizumab ($6.1 billion), and bevacizumab ($3.4 billion); the highest-spending Part D drugs were ibrutinib ($6.6 billion), palbociclib ($1.6 billion), and droxidopa ($0.5 billion; Figure 2). Accelerated approval drugs represented 2.5% of $127 billion estimated total net Part D drug spending in 2019 and 16% of $37 billion total net Part B drug spending.^5,6^

## Discussion

From 2015 through 2019, Medicare spending on drugs with an accelerated approval indication doubled from $4.7 billion to $9.1 billion, with most of this growth among Part B drugs. Our estimates of Part B spending exclude Medicare Advantage beneficiaries, and our findings may not predict future spending if the accelerated approval pathway is used to approve high-cost drugs that treat large patient populations (as happened in 2021 with aducanumab for Alzheimer disease).

Only 13% of Medicare spending on accelerated approval drugs occurred while these drugs were exclusively marketed under the accelerated approval pathway, while most spending was on drugs with multiple indications approved via both accelerated and traditional FDA pathways. This poses a challenge for policy makers seeking to lower Medicare spending on drugs with unproven benefit, since Medicare neither reports indication-specific drug spending nor reimburses drugs differently based on patients’ diagnoses. While accelerated approval drugs are approved based on pivotal trials assessing surrogate measures, some may offer more benefits to patients than others. If the goal of policy makers is to better align Medicare drug spending with clinical value, focusing on accelerated approval drugs alone will be insufficient.

## Notes

Supplement. eMethods. eTable 1. Class-specific estimates of Medicare Part D discounts eTable 2. Estimated Medicare Part B spending in the years immediately after launch
