# Supplementary material for: Medicare Spending on Drugs With Accelerated Approval, 2015-2019
Source: JAMA Health Forum. 2021 Dec 3;2(12):e213937. doi: 10.1001/jamahealthforum.2021.3937 (PMC8796889; doi:10.1001/jamahealthforum.2021.3937)
Supplement: Supplement. — eMethods. eTable 1. Class-specific estimates of Medicare Part D discounts eTable 2. Estimated Medicare Part B spending in the years immediately after launch [file jamahealthforum-e213937-s001.pdf]

Supplemental Online Content

Rome BN, Feldman WB, Kesselheim AS. Medicare spending on drugs with accelerated approval, 2015-2019. *JAMA Health Forum.* 2021;2(12):e213937.
doi:10.1001/jamahealthforum.2021.3937

eMethods.

eTable 1. Class-specific estimates of Medicare Part D discounts
eTable 2. Estimated Medicare Part B spending in the years immediately after launch

This supplemental material has been provided by the authors to give readers additional information about their work.

© 2021 Rome BN et al. *JAMA Health Forum.*

eMethods

We used a published US Food and Drug Administration (FDA) list to identify all drugs with an accelerated approval indication from 1991 (the program's inception) through December 2019.[1] We excluded accelerated approvals that were converted to full approvals or withdrawn before January 2015 and drugs that had no documented spending in the Medicare Part B or D Drug Spending Dashboards.

For each remaining drug, we reviewed the FDA regulatory history to identify whether the drug was approved for additional indications via the traditional FDA approval pathway. If so, we recorded the time from January 2015 – December 2019 when a drug's labelling exclusively included accelerated approval indications and time when the labeling contained indications approved via both accelerated and traditional approval pathways.

For example, venetoclax (Venclexta) was initially granted accelerated approval to treat chronic lymphocytic leukemia in April 2016, and this indication was converted to traditional FDA approval in June 2018. The drug was granted a second accelerated approval to treat acute myeloid leukemia in November 2018, which was not converted to full approval until after December 2019. Based on this, venetoclax was exclusively approved via accelerated approval from April 2016 – June 2018 and non-exclusively approved for accelerated approval from November 2018 – December 2019.

From 2015-2019, the 66 drugs in our cohort had a median of 1.3 years (IQR: 0—3.2 years) of exclusive labeling for accelerated approval indications and 2.9 years (IQR: 1.3—5 years) with any accelerated approval indication on the labeling.

© 2021 Rome BN et al. *JAMA Health Forum.*

Medicare Part D Rebates

Reported Part D drug spending includes spending for Medicare beneficiaries in standalone Part D plans and Medicare Advantage prescription drug plans, but it does not account for confidential manufacturer rebates and coverage gap discounts.[2] We estimated average annual discounts for brand-name drugs in Medicare Part D based on our prior published methodology.[3] We identified average rebates for specific drug classes in 2016 based on a recent Government Accountability Office report.[4] We then added the average coverage gap discounts in 2016 (5.1% of gross brand-name spending) to class-specific rebates. We estimated class-specific estimates in other years by assuming that class-specific estimates grew at the same rate as overall discounts in Medicare Part D (eTable 1).

eTable 1: Class-specific estimates of Medicare Part D discounts

	Estimated brand-name discounts (%)				
**Drug Class**	**2015**	**2016**	**2017**	**2018**	**2019**
Endocrine/Diabetes	44.9	46.2	48.6	52.2	53.8
Infectious Disease	23.6	24.9	27.3	30.9	32.5
Respiratory	35.9	37.2	39.6	43.2	44.8
Neurologic	26.3	27.6	30.0	33.6	35.2
Oncologic	5.8	7.1	9.5	13.1	14.7
Cardiovascular	42.1	43.4	45.8	49.4	51.0
Immunologic	7.9	9.2	11.6	15.2	16.8
Blood agent	30.6	31.9	34.3	37.9	39.5
Genitourinary	43.3	44.6	47.0	50.6	52.2
Musculoskeletal	20.6	21.9	24.3	27.9	29.5
Gastrointestinal	50.3	51.6	54.0	57.6	59.2
Ophthalmologic	49.3	50.6	53.0	56.6	58.2

In a sensitivity analysis, we alternatively estimated drug-specific annual Part D discounts based on average quarterly non-Medicaid rebates reported by SSR Health.[5] These rebates are not specific to Medicare Part D and on average tend to overestimate average Medicare Part D rebates.[3] SSR Health rebate estimates were available in at least one year for 39 (59%) of the 66 drugs in our cohort; for the remainder, we assumed no rebates.

© 2021 Rome BN et al. *JAMA Health Forum.*

Medicare Part B Adjustments

Reported spending in the Medicare Part B dashboard has two limitations. First, unlike the reported Part D spending, Medicare Part B spending data exclude spending among Medicare Advantage beneficiaries. Medicare Advantage represents a growing share of total Medicare beneficiaries, increasing from 31.5% in 2015 to 35.5% in 2019.[6] For these beneficiaries, plans do not publicly report use and spending on clinician-administered drugs or other health care services. As a result, our spending estimates for Part B drugs substantially underestimate total Medicare spending on these drugs.

Second, the Part B dashboard only includes spending data for drugs with a Healthcare Common Procedure Coding System (HCPCS) code, which is often not assigned until 1-2 years after a drug is marketed. As a result, there were 9 drugs with missing Part B spending data immediately following product launch but before spending could be reported in the Part B Drug Spending Dashboard.[7] We estimated Part B spending in these missing years based on spending in the first reported year, proportional to annual US net sales of each drug obtained from SSR Health (**eTable 2**).[5] For example, pembrolizumab was launched in September 2014 but the first Medicare Part B spending was reported in 2016 ($326 million, not adjusted for inflation). Meanwhile, pembrolizumab had annual net US sales of $393 million in 2015 and $752 million in 2016 (i.e. sales in 2015 were 52% of sales in 2016). We used this proportion to estimate $171 million Medicare Part B spending on pembrolizumab in 2015 (52% of $326 million). This approach assumed that Medicare Part B represented a stable proportion of US net spending over time. US net sales were unavailable for two drugs (Avelumab and Belinostat), so we did not estimate missing spending years for these drugs.

© 2021 Rome BN et al. *JAMA Health Forum.*

eTable 2: Estimated Medicare Part B spending in the years immediately after launch

			Annual Net Sales (Millions USD) [a]			Medicare Part B Spending (Millions USD) [b]		
**Drug (Brand Name)**	**Market Launch**	**Year X (First Year Medicare Part B Spending Reported)**	**Year X**	**Year X-1** (% of Year X)	**Year X-2** (% of Year X)	**Year X** (Reported)	**Year X – 1** (Estimated)	**Year X – 2** (Estimated)
Atezolimumab (Tecentriq)	May 2016	2018	480	464 (97%)	156 (32%)	241	233	78
Blinatumomab (Blincyto)	Dec 2014	2016	85	3 (4%)	-	6	0.2	-
Daratumumab (Darzalex)	Nov 2015	2017	884	484 (55%)	20 (2%)	676	370	15
Durvalumab (Imfinzi)	May 2017	2019	1,041	564 (54%)	19 (2%)	719	390	13
Nivolumab (Opdivo)	Dec 2014	2016	2,664	823 (31%)	-	1,220	377	-
Olaratumab (Lartruvo)	Oct 2016	2018	191	162 (84%)	11 (6%)	54	46	3
Pembrolizumab (Keytruda)	Sep 2014	2016	752	393 (52%)	-	326	171	-

[a] Obtained from SSR Health, which collects quarterly net sales data from public manufacturer financial reports. Net sales are not adjusted for inflation.
[b] Medicare Part B spending in Year X comes from the Medicare Part B Drug Spending Dashboard, and spending in prior years is estimated by multiplying spending in Year X by the percent of annual net sales in years (X-1) or (X-2) to Year X (e.g. 241 x 97% = 233).

© 2021 Rome BN et al. *JAMA Health Forum.*

References

1. US Food and Drug Adminstration (FDA). *Accelerated Approvals*.; 2021. Accessed April 14, 2021. https://www.fda.gov/drugs/nda-and-bla-approvals/accelerated-approvals

2. Centers for Medicare & Medicaid Services. *Medicare Part D Drug Spending Dashboard & Data*.; 2019. Accessed May 11, 2021. https://www.cms.gov/Research-Statistics-Data-and-Systems/Statistics-Trends-and-Reports/Information-on-Prescription-Drugs/MedicarePartD.html

3. Feldman WB, Rome BN, Raimond VC, Gagne JJ, Kesselheim AS. Estimating Rebates and Other Discounts Received by Medicare Part D. *JAMA Health Forum*. 2021;2(6):e210626. doi:10.1001/jamahealthforum.2021.0626

4. US Government Accountability Office. *Use of Pharmacy Benefit Managers and Efforts to Manage Drug Expenditures and Utilization*.; 2019. https://www.gao.gov/products/gao-19-498

5. SSR Health. *US Brand Rx Net Pricing Tool*.; 2021. Accessed April 13, 2021. http://www.ssrhealth.com/us-brand-rx-net-price-tool/

6. Boards of Trustees of the Federal Hospital Insurance and Federal Supplementary Medical Insurance Trust Funds. *2020 Annual Report of the Boards of Trustees of the Federal Hospital Insurance and Federal Supplementary Medical Insurance Trust Funds*.; 2020. https://www.cms.gov/files/document/2020-medicare-trustees-report.pdf

7. Centers for Medicare & Medicaid Services. *Medicare Part B Drug Spending Dashboard*.; 2019. Accessed May 11, 2021. https://www.cms.gov/Research-Statistics-Data-and-Systems/Statistics-Trends-and-Reports/Information-on-Prescription-Drugs/MedicarePartB

© 2021 Rome BN et al. *JAMA Health Forum.*
